# Effect of Lung-Protective Ventilation on Changes in Regional Cerebral Oxygen Saturation During Thoracoscopic Surgery in Children: A Prospective, Single-Center, Randomized Controlled Trial

**DOI:** 10.3390/children13091243

**Published:** 2026-09-14

**Authors:** Guoliang Liu, Fang Wang, Lijing Li, Tiehua Zheng, Zhengzheng Gao, Jianmin Zhang

**Affiliations:** Department of Anesthesiology, Beijing Children’s Hospital, Capital Medical University, National Center for Children’s Health, Beijing 100045, China; liuguoliang0@163.com (G.L.);

**Keywords:** one-lung ventilation, regional cerebral oxygen saturation, lung-protective ventilation, pediatrics, postoperative complications

## Abstract

**Highlights:**

**What are the main findings?**

- For children undergoing thoracoscopic surgery with one-lung ventilation, lung-protective ventilation (4–6 mL/kg tidal volume plus 5 cmH_2_O PEEP) was associated with a markedly lower rate of intraoperative cerebral desaturation (≥20% fall in regional cerebral oxygen saturation), relative to conventional ventilation (8–10 mL/kg tidal volume, no PEEP).
- Patients receiving lung-protective ventilation exhibited higher nadir regional cerebral oxygen saturation values. Desaturation events decreased by more than 60% (14.6% versus 37.5%). Post-operative pulmonary complication rates fell by over half (14.6% versus 33.3%), and no pneumothorax cases occurred in this group.
- Median postoperative length-of-stay was one-day shorter in the lung-protective ventilation arm (4.0 versus 5.0 days), reflecting faster clinical recovery.

**What is the implication of the main finding?**

- Low-tidal-volume ventilation (4–6 mL/kg during one-lung ventilation) was clinically feasible and well-tolerated. It maintained adequate surgical exposure without safety concerns, lending support for considering this protective strategy for pediatric one-lung-ventilation procedures.
- This work demonstrates the potential value of near-infrared spectroscopy to detect cerebral deoxygenation missed by pulse oximetry. The findings indicate that combined use of this monitor with lung-protective ventilation warrants further evaluation for optimizing paediatric perioperative care.

**Abstract:**

**Background:** Low regional cerebral oxygen saturation (rScO_2_) in children is linked to neurological sequelae. Lung-protective ventilation (LPV) mitigates lung injury but its effect on intraoperative rScO_2_ is uncertain. Methods: One hundred and four children (<6 years) undergoing thoracoscopic mediastinal mass resection or lobectomy were randomized to LPV (n = 52; tidal volume: 6 mL/kg double-lung ventilation [DLV], 4 mL/kg one-lung ventilation [OLV] + 5 cm H_2_O PEEP) or Control (n = 52; 10 mL/kg DLV, 8 mL/kg OLV + zero PEEP). rScO_2_ was monitored continuously. Primary outcome was incidence of low rScO_2_ during OLV (≥20% decrease from baseline ≥1 min). Secondary outcomes included lowest rScO_2_, postoperative pulmonary complications (PPCs), and hospital length of stay (LOS). Results: Ninety-six children were analyzed (n = 48/group). Overall low rScO_2_ incidence during OLV was 25/96 (26%). Significantly low rScO_2_ incidence occurred with LPV (7/48, 14.6%) versus control (18/48, 37.5%; *p* = 0.011). The lowest intraoperative rScO_2_ was significantly higher in the LPV group 76.5 (70.3, 80.0) compared to control 70.5 (60.3, 76.0); *p* < 0.001. PPC incidence was lower with LPV (7/48, 14.6%) than control (16/48, 33.3%; *p* = 0.031). Postoperative LOS was shorter in the LPV group 4.0 [4.0–5.0] days versus control group 5.0 [4.3–6.0] days, *p* < 0.001. Conclusions: Compared to conventional ventilation, an LPV strategy significantly reduces the incidence of low rScO_2_ during OLV in young children undergoing thoracoscopic surgery. LPV also decreases PPCs and shortens hospital stay. Clinical trial registration: ChiCTR2400092732.

## 1. Introduction

OLV represents the predominant airway management strategy for pediatric thoracoscopic surgery, offering optimal surgical field exposure. However, this technique carries inherent limitations, most notably the risks of intraoperative hypoxemia and postoperative pulmonary complications [1,2,3]. As a metabolically active organ with high oxygen demand, the brain exhibits heightened sensitivity to hypoxic conditions [4]. Diminished cerebral oxygenation has been associated with potential neurological alterations and developmental impairments [5,6,7]. Consequently, hypoxemia during OLV poses a significant threat of cerebral injury. Early detection and timely intervention for intraoperative cerebral hypoxia are crucial for mitigating associated adverse neurological outcomes.

Compared to conventional peripheral oxygen saturation (SpO_2_) monitoring, near-infrared spectroscopy (NIRS) has emerged as a valuable tool for the early detection of cerebral hypoxia [8]. Evidence indicates that changes in cerebral oxygenation detected by NIRS precede alterations observed through pulse oximetry [9]. This non-invasive technology measures rScO_2_ by applying the Beer–Lambert law, quantifying light absorption by oxygenated and deoxygenated hemoglobin within specific near-infrared wavelengths to compute tissue oxygenation in the underlying cerebral cortex [10].

Historically, rScO_2_ monitoring found primary application in neonatal and premature infant intensive care [11] and pediatric congenital heart surgery [12]. Its utilization has subsequently expanded to other pediatric surgical contexts, including thoracoscopic procedures requiring OLV. Notably, adult-based studies have reported that declines in regional cerebral oxygen saturation (rScO_2_) may trigger postoperative cognitive dysfunction even when pulse oxygen saturation (SpO_2_) remains above 90% during one-lung ventilation (OLV) [13]. Nevertheless, few reports have addressed the relevance of this finding for children undergoing one-lung-ventilation surgery. Furthermore, intraoperative rScO_2_ desaturation has been correlated with an increased incidence of postoperative pulmonary complications in children undergoing OLV-dependent thoracoscopic surgery [14].

Therefore, monitoring cerebral oxygenation during OLV is of paramount importance. Concurrently, refining ventilation strategies to minimize both intraoperative hypoxemia and postoperative pulmonary complications is essential. Lung-protective ventilation (LPV), characterized by low tidal volumes, positive end-expiratory pressure (PEEP), and recruitment maneuvers, presents a promising approach. LPV has demonstrated efficacy in reducing postoperative pulmonary complications and maintaining adequate intraoperative oxygenation [3]. Nevertheless, the impact of LPV on rScO_2_ dynamics during pediatric OLV remains inadequately characterized.

We hypothesized that a lung-protective OLV strategy would reduce the incidence of intraoperative cerebral oxygen desaturation, thereby ensuring sustained intraoperative oxygenation and attenuating postoperative pulmonary complications in this population.

## 2. Materials and Methods

### 2.1. Study Population

This prospective randomized controlled trial was approved by the Medical Ethics Committee of Beijing Children’s Hospital, Capital Medical University, China (Approval No.: 2024-Y-183-D) and registered at the Chinese Clinical Trial Registry (ChiCTR2400092732). The study was conducted in accordance with the ethical standards of the Declaration of Helsinki (1964) and its subsequent amendments. Prior to each surgery, the attending anesthesiologist met with the parents/guardians, explained the study protocol, and obtained written informed consent. This trial was registered prior to patient recruitment. The primary outcome, analysis population and core statistical plan were consistent with the prospectively registered protocol. No modifications to primary outcome, analytical population or statistical plan were implemented after trial registration and enrolment commencement.

### 2.2. Inclusion Criteria

Children scheduled for elective thoracoscopic mediastinal mass resection or lobectomy at the Beijing Children’s Hospital, Capital Medical University, National Center for Children’s Health between December 2024 and December 2025 were eligible if they met all the following criteria:

1. Age < 6 years;
2. American Society of Anesthesiologists (ASA) physical status I or II;
3. No respiratory infection within the preceding 2 weeks;
4. Absence of major systemic diseases;
5. Normal preoperative laboratory investigations;
6. Planned OLV using an endobronchial blocker.

### 2.3. Exclusion Criteria

Patients were excluded for any of the following:

1. Preoperative room-air SpO_2_ < 95%;
2. Skin lesions or rashes at cerebral oximetry sensor sites;
3. Conversion to open thoracotomy during surgery;
4. OLV duration < 30 min;
5. Blood products need to be infused;
6. Incomplete intraoperative data.

### 2.4. Randomization and Blinding

Patients were randomly assigned to either the LPV group or the control group using computer-generated simple randomization. An independent anesthesia nurse prepared sequentially numbered, sealed opaque envelopes with a 1:1 allocation ratio. Patients, guardians, surgeons, nursing staff, and outcome assessors were blinded to group assignment. Due to the nature of the intervention, the anesthesiologists performing ventilation management were not blinded.

### 2.5. Study Protocol

All children received general anesthesia in the operating room with continuous monitoring of heart rate (HR), non-invasive blood pressure (BP), SpO_2_, bispectral index (BIS), and core temperature. Near-infrared spectroscopy monitors (NIRS, CAS Medical Systems Inc., Branford, CT, USA) were placed bilaterally on the forehead before anesthesia initiation. The baseline rScO_2_ value was defined as the averaged value of bilateral rScO_2_ readings obtained after preoxygenation, after the children fell asleep following intravenous remimazolam 0.1 mg/kg (Jiangsu Hengrui Pharmaceuticals Co., Ltd., Lianyungang, China) sedation and before further anesthesia-induction agents were administered. Readings from the left and right cerebral hemispheres were averaged for subsequent primary-outcome analysis. For the definition of cerebral desaturation event (rScO_2_ decrease ≥20% relative to baseline), only a drop exceeding the threshold in the averaged bilateral rScO_2_ value was counted as an event; isolated unilateral-hemisphere desaturation without reduction in the bilateral average was not regarded as a cerebral desaturation event. Real-time rScO_2_ data were masked from surgical, anesthesia, and nursing teams as they were collected solely for research purposes.

## 3. Anesthesia Protocol

### 3.1. Induction and Maintenance

Anesthesia was induced with intravenous propofol (3 mg/kg) (Xi’an Libang Pharmaceutical Co., Ltd.,Xi’an, China), sufentanil (0.3 ug/kg) (Yichang Humanwell Pharmaceutical Co., Ltd., Yichang, China), and rocuronium (0.6 mg/kg) to facilitate tracheal intubation. Invasive arterial blood pressure (ABP) and end-tidal carbon dioxide (EtCO_2_) were continuously monitored. Anesthesia maintenance was achieved with propofol infusion (8–10 mg/kg/h) and remifentanil (Yichang Humanwell Pharmaceutical Co., Ltd., Yichang, China) infusion (0.2–0.4 ug/kg/min). Remifentanil dosing was titrated to maintain HR and mean arterial pressure (MAP) within ±20% of baseline values. Hypotension exceeding 20% below baseline was treated with vasopressors, while bradycardia (>20% decrease) was managed with atropine.

### 3.2. One-Lung Ventilation Technique

OLV was established using a 5F Arndt endobronchial blocker (Cook Medical Inc., Bloomington, IN, USA). The blocker was positioned externally to the tracheal tube, with tracheal intubation performed using a tube 0.5 mm smaller than standard sizing. Under fiberoptic bronchoscopic guidance, the blocker was advanced into the main bronchus of the operative lung. The blocker balloon remained deflated during initial DLV. Following tracheal intubation, an arterial catheter was inserted for hemodynamic monitoring. After positioning the patient laterally, the blocker balloon was inflated to initiate OLV.

### 3.3. Ventilation Strategies

For children younger than six years, predicted body weight (PBW) was calculated by the formula: predicted body weight (kg) = (age + 4) × 2. Tidal volume settings for lung-protective ventilation were calculated according to PBW. Patients in the control group received conventional ventilation with tidal volume of 8–10 mL/kg without PEEP. This conventional setting represents a historical comparator for pediatric one-lung ventilation described in pediatric anesthesia textbooks and clinical literature prior to broad implementation of lung-protective ventilation. The lung-protective ventilation (LPV) intervention combined reduced tidal volume and applied PEEP simultaneously; therefore, individual effects of tidal-volume reduction and PEEP could not be separated in the present study.

Control Group: DLV: Tidal volume (Vt) = 10 mL/kg predicted body weight (PBW); OLV: Vt = 8 mL/kg PBW; Zero PEEP.

Lung-Protective Ventilation Group: DLV: Vt = 6 mL/kg PBW; OLV: Vt = 4 mL/kg PBW; PEEP = 5 cm H_2_O.

Both groups received DLV at a respiratory rate (RR) of 20–25 breaths/min. During OLV, RR was increased as needed to maintain EtCO_2_ between 35 and 45 mmHg. Fraction of inspired oxygen (FiO_2_) was set at 0.5 during DLV and 1.0 during OLV, with an inspiratory-to-expiratory (I:E) ratio of 1:2. The respiratory rate had a predefined target range; however, it could be increased beyond this range as a rescue clinical maneuver in the setting of intraoperative hypercapnia or inadequate ventilation. There was no absolute protocol-fixed PaCO_2_ or pH threshold that compelled mandatory ventilation adjustment. Ventilator modifications were determined by the attending anesthesiologist integrating hemodynamic status and surgical requirements. Clinically active intervention was strongly considered when PaCO_2_ exceeded 65 mmHg or pH decreased below 7.25.

Severe hypoxemia (SpO_2_ < 90%) during OLV prompted conversion to DLV with increased FiO_2_ and oxygen flow. Following tumor resection or lobectomy, the blocker balloon was deflated to resume DLV. A standardized lung recruitment maneuver was performed post-OLV: airway pressure sustained at 30 cm H_2_O for 15–20 s [15].

### 3.4. Fluid Management and Recovery

All patients received crystalloid solutions guided by goal-directed fluid-therapy principles to avoid volume overload. Blood products were administered based on intraoperative blood loss. Tracheal extubation was performed upon surgery completion, with patients transferred to the post-anesthesia care unit (PACU). Postoperative laboratory tests, imaging data, and vital signs were collected until hospital discharge.

### 3.5. Data Collection

rScO_2_, MAP, HR, SpO_2_, peak airway pressure (Peak), plateau pressure (Plat), Vt, RR, EtCO_2_ were recorded at the following time points: T1, after sedation in the operating room; T2, after commencement of two lung ventilation; T3, at the beginning of one lung ventilation; T4, 30 min after commencement of one lung ventilation; T5, at the end of one lung ventilation; T6, at the end of surgery. Driving pressure (ΔP = Plat − PEEP) was calculated. Arterial blood gas (ABG) analysis was performed at T3, T5, and T6. Lung collapse status was assessed at T3 and T4. The degree of lung collapse was evaluated by a surgeon blinded to group allocation using a verbal rating scale (Lung Collapse Scale: 0 = no collapse to 10 = complete collapse) [16]. Postoperative pulmonary complications occurring during the hospital stay and postoperative hospital length of stay (LOS) were recorded. Pulmonary complications included pneumothorax, atelectasis, postoperative hypoxemia, and respiratory tract infection. Postoperative pulmonary complications were defined with pre-specified criteria: pneumothorax was confirmed by the presence of intra-pleural air on chest radiograph; atelectasis was defined as radiographic pulmonary collapse accompanied by diminished breath sounds or increased oxygen demand; Respiratory tract infections required new-onset fever, purulent airway secretions and new pulmonary infiltrates on imaging; postoperative hypoxemia was defined as SpO_2_ < 95% on room air sustained for more than 30 min within 24 h post-operatively. Post-operative chest radiography was routinely obtained 2–4 h after admission to the post-anesthesia care unit. Evaluation of complications followed a standardized case-report-form-driven protocol. Outcome assessors were blinded to group assignment.

### 3.6. Outcome

The primary outcome was the incidence of intraoperative cerebral desaturation during OLV. Cerebral desaturation was defined as a decrease in rScO_2_ of ≥20% from baseline values persisting for ≥1 min [7].

Secondary outcomes included the intraoperative minimum rScO_2_ and minimum SpO_2_; changes in rScO_2_ and vital signs at each time point (T1–T6); ventilation parameters and ABG results; the incidence of postoperative pulmonary complications; and postoperative hospital LOS.

### 3.7. Sample Size Estimation

Based on previous adult studies reporting an incidence of cerebral desaturation (rScO_2_ decrease ≥20% from baseline) as high as 70% in thoracic surgery patients undergoing OLV [17], we hypothesized that LPV would reduce this incidence by 50% (to 35%). Using PASS software, with a power (1-β) of 0.9 and a two-sided significance level (α) of 0.05, 41 patients per group were required for this two-group study. Accounting for an estimated 20% dropout rate, a total of 104 children were planned for enrollment. Of note, pediatric data regarding the baseline incidence of cerebral desaturation during one-lung ventilation were scarce at study design; therefore, the initial 70% event rate assumption was derived from the literature pertaining to adult patients. After enrollment was completed, the actual incidence of cerebral desaturation in the pediatric control group was only 37.5%, which was substantially lower than the adult-based assumption. This lower observed event rate may have reduced the actual statistical power of the present study, and this limitation has been further discussed in the Section 5.

### 3.8. Statistical Analysis

Statistical analyses were performed using SPSS for Windows (Version 23.0; SPSS Inc., Chicago, IL, USA). Statistical analysis was performed based on the per-protocol (PP) dataset. The normality of data distribution was assessed using the Kolmogorov–Smirnov test. Categorical variables are presented as numbers and percentages. Continuous variables are expressed as mean ± standard deviation (SD) or median (interquartile range [IQR]), as appropriate. Group comparisons for categorical data were performed using the Chi-square (χ^2^) test. For continuous data, the Student’s *t*-test or the Mann–Whitney U test was used. Repeated-measures analysis of variance was used to compare repeatedly measured intraoperative variables across time points between the two groups. Mauchly’s test was used to assess the sphericity assumption. Greenhouse–Geisser correction was adopted when the sphericity assumption was violated. For post hoc between-group comparisons at individual time-points, the Bonferroni correction was performed to control type-I errors arising from multiple comparisons. Partial eta-squared was reported as the effect-size estimate. A *p*-value < 0.05 was considered statistically significant.

## 4. Results

### 4.1. Patient Characteristics

**Figure 1:**
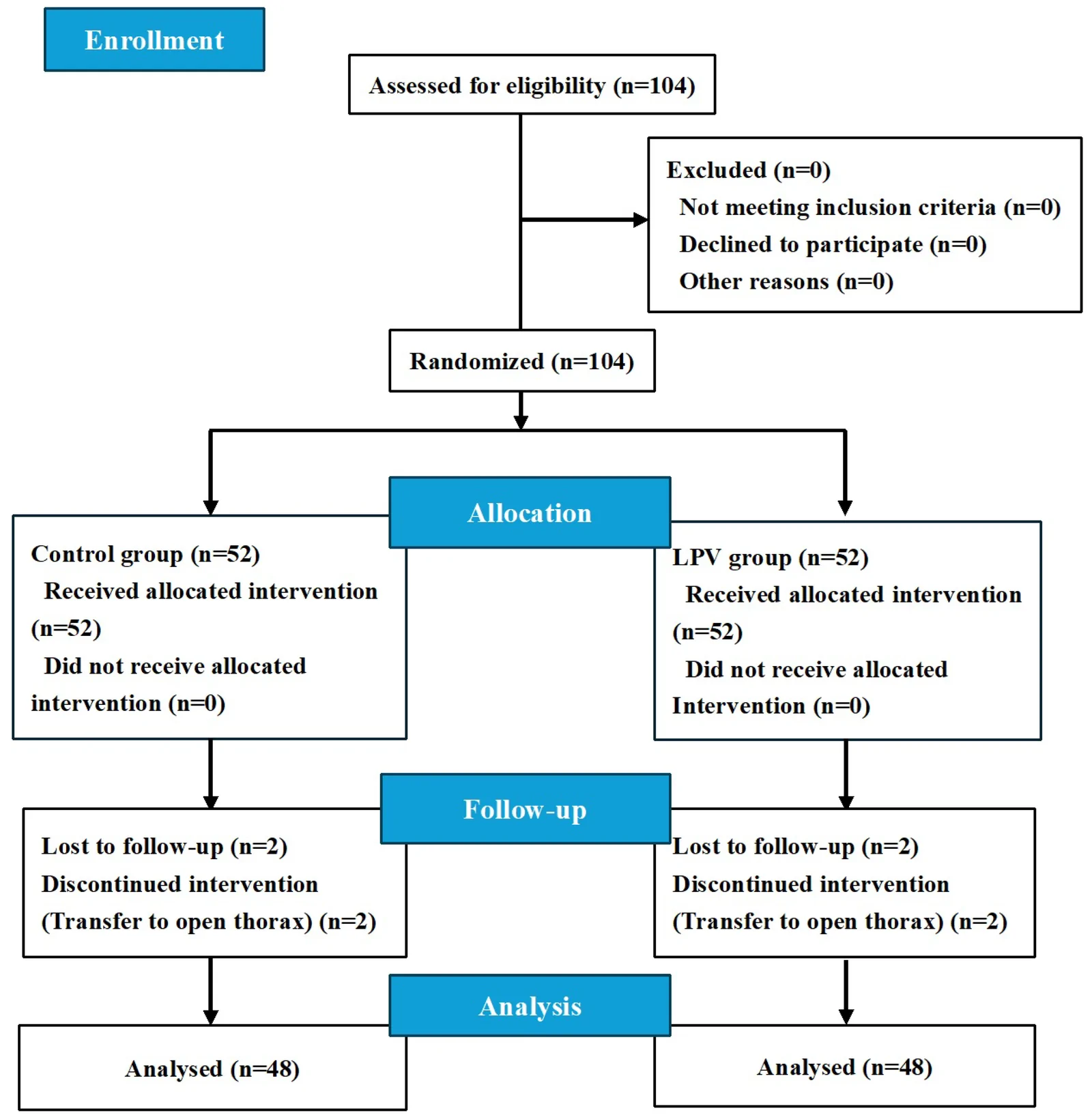
CONSORT diagram.CONSORT, Consolidated Standards of Reporting Trials; LPV, lung protective ventilation.

Between December 2024 and December 2025, a total of 104 pediatric patients were enrolled and randomly assigned to the two study groups. Two patients in the control group and two in the LPV group were subsequently excluded due to conversion to thoracotomy. An additional four patients (two per group) were excluded because of missing data resulting from malfunction of the cerebral oximetry device. Consequently, data from the remaining 96 children (48 per group; control and LPV) were included in the final analysis (Figure 1). Baseline demographic and intraoperative characteristics are summarized in Table 1. All patients were successfully extubated at the conclusion of the procedure and transferred to the PACU.

### 4.2. Primary Outcome

Cerebral desaturation (rScO_2_ reduction ≥20% from baseline) during one-lung ventilation was observed in 25 of 96 children (26.0%). The incidence was significantly higher in the control group compared to the LPV group (18/48 [37.5%] vs. 7/48 [14.6%]; *p* = 0.011).

### 4.3. Secondary Outcomes

Lowest rScO_2_ and SpO_2_ Values.

During OLV, the minimum rScO_2_ was significantly lower in the control group than in the LPV group (70.5 [60.3–76.0] vs. 76.5 [70.3–80.0]; *p* < 0.001). No significant intergroup difference was observed in minimum SpO_2_ (Table 1).

### 4.4. Temporal Changes in Vital Signs and rScO_2_

Repeated-measures analysis of variance was performed. Mauchly’s test indicated a violation of sphericity assumption (*p* < 0.05); therefore, the Greenhouse–Geisser correction was applied. There was a significant group-by-time interaction for regional cerebral oxygen saturation (F = 4.489, *p* = 0.002, partial η^2^ = 0.049), suggesting divergent temporal patterns between the two groups. Subsequent post hoc between-group comparisons at each time-point were conducted, with *p*-values adjusted using the Bonferroni method for multiple testing. Statistically significant intergroup differences were observed at T5 (mean difference: 3.81, 95%CI 1.66–5.96, adjusted *p* = 0.004), while no significant differences were detected at other time points.

Repeated-measures analysis of variance revealed no significant group-by-time interactions for BIS, MAP, HR, and SpO_2_. Post hoc between-group comparisons at each time point, with *p*-values adjusted by the Bonferroni method for multiple testing, showed no statistically significant intergroup differences across all time points (Table 2).

### 4.5. Ventilation Parameters and Blood Gas Analysis

Compared to the LPV group, the control group demonstrated significantly higher values for tidal volume, driving pressure (ΔP), and peak airway pressure (*p* < 0.05). Conversely, respiratory rate and end-tidal carbon dioxide were elevated in the LPV group relative to controls (*p* < 0.05).

Arterial blood gas analysis showed that in the lung-protective ventilation (LPV) group, the partial pressure of carbon dioxide (PaCO_2_) was significantly increased and the pH value was significantly decreased at time points T5 and T6 (*p* < 0.05). In the LPV group, at the time-point of peak PaCO_2_ (63 ± 17 mmHg), the corresponding pH was 7.18 ± 0.09. Clinically important hypercapnia (PaCO_2_ > 65 mmHg accompanied by pH < 7.25) occurred in 16 patients within this group. Most hypercapnia episodes were transient and resolved with ventilator adjustment during surgery. No significant intergroup differences were observed in partial pressure of oxygen or hemoglobin levels at T3, T5, or T6. Similarly, lung collapse scores were not significantly different between groups at both T3 and T4 (Table 3).

### 4.6. Postoperative Pulmonary Complications and Hospital Stay

The overall incidence of postoperative pulmonary complications (PPCs) was 24.0% (23/96) among all participants. Respiratory tract infections accounted for 17.7% of complications (17/96), while pneumothorax occurred in 6.3% of cases (6/96). No severe complications were observed. Respiratory tract infections resolved with antibiotic therapy prior to discharge, and pneumothoraces resolved spontaneously without intervention.

The incidence of PPCs was significantly higher in the control cohort compared to the LPV group (33.3% [16/48] vs. 14.6% [7/48]; *p* = 0.031) (Table 4). Postoperative length of hospital stay was prolonged in controls relative to the LPV group (median [IQR]: 5.0 [4.3–6.0] days vs. 4.0 [4.0–5.0] days; *p* < 0.001).

## 5. Discussion

This prospective randomized controlled trial provides the first evidence demonstrating that a lung-protective ventilation strategy (tidal volume 4–6 mL/kg + PEEP 5 cmH_2_O) significantly reduces the incidence of cerebral desaturation events (defined as rScO_2_ decrease ≥20% from baseline) during one-lung ventilation for thoracoscopic surgery in children under 6 years (14.6% vs. 37.5%, *p* = 0.011). Concurrently, implementation of LPV was associated with a lower rate of postoperative pulmonary complications (PPCs) (14.6% vs. 33.3%, *p* = 0.031) and a reduced length of postoperative hospital stay (median [IQR]: 4.0 [4.0–5.0] days vs. 5.0 [4.3–6.0] days; *p* < 0.001). These findings offer substantial evidence to guide optimization of ventilation management in pediatric OLV.

In adult patients undergoing non-cardiac surgery, a reduction in rScO_2_ exceeding 20% from baseline has demonstrated a significant association with an elevated incidence of postoperative cognitive dysfunction and major cerebral morbidity [18]. Several pediatric studies [19] have also demonstrated that a decline of 20% or greater in regional cerebral oxygen saturation markedly raises the probability of adverse postoperative behavioral changes among children. This threshold may hold greater clinical relevance for pediatric populations due to their reduced tolerance to hypoxic insults. Consequently, in reference to prior pediatric studies [7], we defined an rScO_2_ reduction of ≥20% relative to baseline as the primary endpoint of the present study. Furthermore, thoracic surgical procedures involving OLV in adults are frequently associated with substantial declines in rScO_2_. Conventional parameters employed to guide OLV management in thoracic surgery (such as SpO_2_ or PaO_2_) have proven insufficient for detecting clinically significant cerebral oxygen desaturation [17]. Our findings corroborate this observation: despite comparable peripheral SpO_2_ and arterial PaO_2_ levels between groups, the incidence of cerebral desaturation events was markedly higher under conventional ventilation. This elevated incidence was significantly attenuated by the implementation of LPV.

The significantly lower incidence of cerebral desaturation in the LPV group is likely attributable to possible explanations of synergistic mechanisms:

1. Enhanced Oxygenation and Reduced Shunt: The combination of low tidal volume and PEEP helps preserve functional residual capacity, thereby diminishing intrapulmonary shunt in the non-ventilated lung and improving overall oxygenation efficiency [3]. Although peripheral SpO_2_ remained similar, the consistently higher rScO_2_ values observed in the LPV group (e.g., 88% vs. 84% at T5; *p* < 0.05) suggest superior maintenance of cerebral oxygen delivery.
2. Critical Role of Driving Pressure (ΔP): Evidence suggests that driving pressure-guided ventilation during OLV is correlated with superior perioperative oxygenation, a reduced incidence of PPCs, and improved respiratory system compliance in thoracic surgery patients [20]. In the present study, the LPV group demonstrated significantly lower ΔP at all measured intraoperative time points (*p* < 0.001). This reduction in mechanical stress may contribute to decreased pulmonary vascular resistance, supporting sustained cardiac output and cerebral perfusion [3].
3. Potential Contribution of Permissive Hypercapnia: Permissive hypercapnia has been proposed to enhance oxygenation during OLV without substantially increasing the risk of PPCs or prolonging hospitalization [21]. The moderately elevated arterial PaCO_2_ observed in our LPV cohort at T5 (63 ± 17 mmHg vs. 53 ± 10 mmHg in controls) could augment cerebral blood flow via CO_2_-induced vasodilation [4], potentially contributing to the lower desaturation incidence. Accordingly, better rScO_2_ in the LPV arm cannot be solely attributed to lung-protective ventilation, and hypercapnia is an important confounding variable for cerebral oxygen monitoring during pediatric one-lung ventilation.

Pediatric respiratory physiology, characterized by a smaller functional residual capacity, larger closing volume, and greater chest wall compliance, predisposes infants and young children to atelectasis under general anesthesia and a higher susceptibility to PPCs [3]. Patients undergoing thoracic surgery face additional risks due to potential injury to the ventilated lung during OLV, lung tissue resection, and surgical manipulation of the non-ventilated lung, processes which may incite tissue damage and inflammatory mediator release [22]. The >50% reduction in PPC risk observed with LPV in our study, particularly the absence of pneumothorax in the LPV group compared to an incidence of 6.3% in controls, may be explained by several factors:

1. Alveolar Recruitment Maneuvers: Protocolized lung recruitment (30 cmH_2_O sustained for 15–20 s) performed at the conclusion of surgery likely mitigated atelectasis [15].
2. Reduced Barotrauma Risk: Lower peak airway pressures documented in the LPV group probably decreased the susceptibility to pneumothorax.
3. Attenuation of Ventilator-Induced Lung Injury (VILI): Lower ΔP may suppress the release of inflammatory mediators associated with mechanical lung injury, consequently reducing complications such as pneumonia [23].

The concomitant reduction in postoperative hospital stay further underscores the comprehensive clinical benefits afforded by LPV, aligning well with enhanced recovery after surgery (ERAS) principles.

The overall incidence of cerebral desaturation observed in this pediatric cohort (26%) was notably lower than the 70% reported in adult OLV studies [17], suggesting potential age-specific differences in cerebral oxygenation regulation. Additionally, alterations in rScO_2_ are known to precede changes in SpO_2_ [9]. Our data reveal that significant cerebral desaturation preceded measurable SpO_2_ changes despite maintained hemodynamic stability. This supports NIRS implementation for incipient cerebral hypoxia identification during pediatric thoracic procedures requiring OLV.

While previous pediatric LPV studies often utilized tidal volumes of 6–8 mL/kg [24], our protocol employing 4 mL/kg during OLV provided equivalent surgical field exposure (as evidenced by comparable lung collapse scores), maintained adequate intraoperative oxygenation, and did not incur significant hypercapnia-related risks. For two-lung ventilation (TLV), we set the tidal volume at 6 mL/kg, as supraphysiological tidal volumes above 5–7 mL/kg have been proven to cause ventilator-induced lung injury in children [25,26]. Despite the lung-protective advantages of low-tidal-volume ventilation, this strategy inherently increases the risk of intraoperative atelectasis and poor postoperative pulmonary outcomes [27], which can be effectively offset by standardized PEEP application. Therefore, reasonable PEEP selection is critical to balance lung protection and respiratory hemodynamic stability. Preclinical pediatric animal studies have demonstrated that PEEP levels of 4–8 cm H_2_O can significantly optimize lung compliance [28]. Clinical data from children with acute respiratory failure further showed that PEEP over 6 cm H_2_O improved arterial oxygenation, whereas PEEP exceeding 3 cm H_2_O might impair systemic oxygen transport and cardiac performance [29]. Based on the above evidence and our single-center clinical experience with pediatric patients having normal baseline lung function, a fixed PEEP of 5 cm H_2_O was implemented throughout the LPV protocol in this study.

Pediatric patients administered low-tidal-volume lung-protective ventilation (LPV) demonstrated increased end-tidal and arterial carbon dioxide partial pressures. Such hypercapnic changes are in line with the concept of permissive hypercapnia described in prior clinical studies [30,31]. Importantly, no intraoperative hypoxemia was observed in our cohort, suggesting that low-tidal-volume ventilation supplemented with adequate PEEP is effective in avoiding systemic oxygen desaturation. Collectively, these results indicate that satisfactory gas exchange does not equate to an optimal ventilation strategy. Simply monitoring conventional gas exchange parameters is therefore inadequate for guiding individualized mechanical ventilation. Instead, standardized LPV strategies combining limited tidal volume and appropriate PEEP should be implemented to reduce the risk of postoperative pulmonary complications. Additionally, the mild hypercapnia induced by LPV may enhance hypoxic pulmonary vasoconstriction, which contributes to the alleviation of ventilation-perfusion mismatch during pediatric one-lung ventilation.

## 6. Limitations

1. Single-Center Design: The single-center nature of this study may constrain the generalizability of our findings. Validation of LPV’s applicability in other pediatric surgical contexts requires future investigation through multicenter studies with larger cohorts.
2. Age Range: The relatively broad age range included in our cohort may encompass pediatric patients at differing developmental stages. Future studies should stratify participants into narrower age groups to explore potential age-specific variations in outcomes.
3. The clinical relevance of cerebral desaturation defined as a 20% relative drop in rScO_2_ remains undetermined in children. This threshold is primarily extrapolated from adult data, and we did not conduct postoperative neurological or neurodevelopmental outcome assessments. Consequently, we cannot determine whether detected rScO_2_ declines correspond to real-world neurological harm. Neurological Outcomes: This research focused solely on the incidence of low regional cerebral oxygen saturation (rSO_2_). Further investigation is necessary to determine whether these observed changes in rSO_2_ exert a significant impact on long-term neurological outcomes in this patient population.
4. PEEP Strategy: Our study employed a fixed PEEP level. Given emerging evidence suggesting that individualized PEEP titration may be more beneficial for pediatric patients, a critical future research direction involves examining the correlation between lung-protective ventilation strategies utilizing individualized PEEP and regional cerebral oxygen saturation.
5. We adopted a per-protocol analysis and excluded several randomized patients who were converted to thoracotomy or had incomplete primary-outcome data. Although baseline characteristics were comparable between all randomized patients and the analytical sample, post-randomization exclusion may still introduce potential selection bias. Therefore, our results mainly reflect the effects of lung-protective ventilation in children who successfully completed thoracoscopic one-lung-ventilation according to trial protocol, and may not be generalized to patients requiring intraoperative conversion to open surgery.
6. The sample-size calculation was initially based on event rates reported in adult patients. The real-world incidence of cerebral desaturation among children in our control group was substantially lower than the adult-derived assumption, which may decrease the actual statistical power and limit the ability to detect small-magnitude intergroup differences.
7. The LPV intervention included both reduced tidal volume and PEEP. Owing to the combined-intervention study design, we cannot distinguish the independent effect of lower tidal volume from that of PEEP. Our results should be interpreted as the overall effect of this composite lung-protective ventilation strategy. Future factorial-design studies are warranted to clarify the separate contributions of tidal-volume magnitude and PEEP in children undergoing one-lung ventilation.

## 7. Conclusions

In summary, lung-protective ventilation (tidal volume 4–6 mL/kg + PEEP 5 cmH_2_O) effectively maintains cerebral oxygen supply–demand equilibrium during OLV in children, significantly reducing the occurrence of cerebral desaturation events. This ventilation strategy concurrently lowers the risk of postoperative pulmonary complications and shortens the recovery period.

## Figures and Tables

**Table 1:** Baseline characteristics and intraoperative variables of all patients. Data are presented as mean ± standard deviation, median (inter-quartile range), or n (%). LPV, lung protective ventilation; OLV, one-lung ventilation; SpO_2_, peripheral oxygen saturation; rScO_2_, regional cerebral oxygen saturation.

Parameters	Control Group (n = 48)	LPV Group (n = 48)	*p*-Value
Age (months)	30.0 (10.3, 48.0)	24.0 (11.0, 40.5)	0.723
Weight (kg)	13.0 (9.9, 18.0)	13.8 (9.8, 17.5)	0.786
Height (cm)	90.0 (74.3, 106.0)	93.0 (78.0, 103.0)	0.985
Sex (male)	29 (60.4)	23 (47.9)	0.219
ASA			0.496
I	15 (31.3)	12 (25)	
II	33 (68.8)	36 (75)	
Type of operation			1.000
Mediastinal tumor	18 (37.5)	18 (37.5)	
Congenital cystic disease of lung	30 (62.5)	30 (62.5)	
Surgical side (left)	22 (45.8)	27 (56.3)	0.307
OLV time (min)	66.0 (42.3, 84.0)	68 (39.5, 102.0)	0.690
Total operation time (min)	78.5 (49.8, 102.5)	79.5 (45.5, 116.0)	0.642
Anesthesia time (min)	118.1 ± 46.5)	123.6 ± 46.4	0.565
Extubation time (min)	16 (13, 18)	15 (13,18)	0.757
Total infused fluid (mL)	250 (150, 350)	223 (146, 300)	0.601
Bleeding and urine output (mL)	41 (22, 62)	47 (22, 80)	0.763
Minimum SpO_2_ during OLV (%)	96.5 (92.0, 99.0)	97.0 (92.0, 99.0)	0.813
Minimum rScO_2_ during OLV (%)	70.5 (60.3, 76.0)	76.5 (70.3, 80.0)	<0.001

**Table 2:** Changes in vital signs and cerebral oxygen saturation during surgery, Data are presented as mean ± standard deviation, median (inter-quartile range). LPV, lung protective ventilation; BIS, bispectral index; SpO_2_, peripheral oxygen saturation; rScO_2_, regional cerebral oxygen saturation; HR, heart rate; MAP, mean arterial pressure; T1, after sedation in the operating room; T2, after commencement of two lung ventilation; T3, at the beginning of one lung ventilation; T4, 30 min after commencement of one lung ventilation; T5, at the end of one lung ventilation; T6, at the end of surgery.

Parameters	Control Group (n = 48)	LPV Group (n = 48)	Mean Difference (95% CI)	Adjusted *p*-Value
T1 rScO_2_ (%)	89.00 ± 1.34	88.65 ± 1.69	−0.35 (−0.97, 0.26)	0.517
BIS	80.19 ± 3.72	79.15 ± 3.11	−1.04 (−2.43, 0.35)	0.608
HR (min^−1^)	123.71 ± 15.12	122.15 ± 16.55	−1.56 (−7.99, 4.86)	1.000
MAP (mmHg)	72.73 ± 6.41	71.98 ± 7.77	−0.75 (−3.64, 2.14)	1.000
SpO_2_ (%)	99.52 ± 0.90	99.62 ± 1.04	0.10 (−0.29, 0.50)	1.000
T2 rScO_2_ (%)	88.98 ± 3.16	89.71 ± 1.27	0.73 (−0.25, 1.71)	0.429
BIS	54.90 ± 6.60	55.52 ± 5.60	0.62 (−1.86, 3.11)	1.000
HR (min^−1^)	116.04 ± 20.34	115.81 ± 17.87	−0.23 (−7.99, 7.53)	1.000
MAP (mmHg)	65.52 ± 6.75	67.62 ± 7.18	2.10 (−0.72, 4.93)	0.711
SpO_2_ (%)	99.73 ± 0.64	99.71 ± 0.65	−0.02 (−0.28, 0.24)	1.000
T3 rScO_2_ (%)	85.46 ± 5.91	84.38 ± 5.77	−1.08 (−3.45, 1.28)	0.517
BIS	55.54 ± 6.51	55.75 ± 5.20	0.21 (−2.18, 2.60)	1.000
HR (min^−1^)	115.21 ± 18.53	115.81 ± 17.15	0.60 (−6.63, 7.84)	1.000
MAP (mmHg)	66.50 ± 9.64	63.06 ± 7.09	−3.438 (−6.871, −0.004)	0.298
SpO_2_ (%)	98.94 ± 3.01	99.69 ± 0.90	0.75 (−0.16, 1.66)	0.557
T4 rScO_2_ (%)	80.85 ± 8.05	84.17 ± 6.66	3.31 (0.32, 6.31)	0.153
BIS	52.67 ± 7.24	54.21 ± 5.86	1.54 (−1.13, 4.21)	0.764
HR (min^−1^)	119.21 ± 18.07	121.50 ± 19.65	2.29 (−5.36, 9.94)	1.000
MAP (mmHg)	65.62 ± 9.40	65.23 ± 9.98	−0.40 (−4.32, 3.53)	1.000
SpO_2_ (%)	98.83 ± 2.11	99.44 ± 1.27	0.60 (−0.10, 1.31)	0.557
T5 rScO_2_ (%)	83.29 ± 5.68	87.10 ± 4.89	3.81 (1.66, 5.96)	0.004
BIS	53.19 ± 7.78	56.23 ± 6.38	3.04 (0.16, 5.92)	0.233
HR (min^−1^)	115.40 ± 17.22	117.79 ± 18.92	2.40 (−4.94, 9.73)	1.000
MAP (mmHg)	68.06 ± 9.98	67.21 ± 10.30	−0.85 (−4.96, 3.26)	1.000
SpO_2_ (%)	99.50 ± 1.07	99.62 ± 1.16	0.12 (−0.33, 0.58)	1.000
T6 rScO_2_ (%)	87.31 ± 4.17	88.73 ± 3.10	1.42 (−0.07, 2.91)	0.249
BIS	61.42 ± 4.38	62.83 ± 4.50	1.42 (−0.38, 3.22)	0.608
HR (min^−1^)	113.08 ± 17.59	116.15 ± 17.10	3.06 (−3.97, 10.09)	1.000
MAP (mmHg)	73.75 ± 10.96	70.65 ± 10.69	−3.10 (−7.49, 1.28)	0.711
SpO_2_ (%)	99.81 ± 0.45	99.92 ± 0.28	0.10 (−0.05, 0.26)	0.694

**Table 3:** Intraoperative respiratory parameters and results of arterial blood gas analysis. Data are presented as mean ± standard deviation, median (inter-quartile range). EtCO_2_, end tidal carbon dioxide pressure; LPV, lung protective ventilation; PaCO_2_, arterial partial pressure of carbon dioxide; PaO_2_, arterial partial pressure of oxygen; T2, after commencement of two lung ventilation; T3, at the beginning of one lung ventilation; T4, 30 min after commencement of one lung ventilation; T5, at the end of one lung ventilation; T6, at the end of surgery.

Parameters	Control Group (n = 48)	LPV Group (n = 48)	*p*-Value
T2 Tidal volume (mL)	124 (95, 175)	79 (61, 99)	<0.001
Respiratory rate (min^−1^)	20 (19, 22)	20 (20, 25)	0.002
Peak airway pressure (cm H_2_O)	17 (15, 19)	15 (14, 16)	<0.001
Driving pressure (cm H_2_O)	16 (14, 18)	9 (8, 10)	<0.001
ETCO_2_ (mmHg)	35 (33, 40)	37 (36, 40)	0.062
T3 Tidal volume (mL)	98 (76, 139)	54 (45, 73)	<0.001
Respiratory rate (min^−1^)	25 (23, 27)	30 (26, 32)	<0.001
Peak airway pressure (cm H_2_O)	27 (24, 29)	21 (20, 24)	<0.001
Driving pressure (cm H_2_O)	26 (22, 28)	15 (13, 18)	<0.001
EtCO_2_ (mmHg)	39 (34, 43)	42 (37, 45)	0.087
Lung collapse score	9.0 (9.0, 9.8)	9.0 (9.0, 9.0)	0.390
PaO_2_ (mmHg)	389 ± 119	380 ± 128	0.730
PaCO_2_ (mmHg)	42 ± 6	43 ± 6	0.225
Hemoglobin (g/L)	109 ± 13	107 ± 10	0.487
PH values	7.30 ± 0.04	7.29 ± 0.04	0.143
T4 Tidal volume (mL)	99 (68, 132)	54 (46, 71)	<0.001
Respiratory rate (min^−1^)	25 ± 3	30 ± 4	<0.001
Peak airway pressure (cm H_2_O)	29 (26, 31)	22 (21, 25)	<0.001
Driving pressure (cm H_2_O)	28 (24, 30)	16 (15, 19)	<0.001
EtCO_2_ (mmHg)	45 ± 10	55 ± 12	<0.001
Lung collapse score	10 (10, 10)	10 (10, 10)	0.317
T5 Tidal volume (mL)	101 (86, 168)	78 (63, 94)	<0.001
Respiratory rate (min^−1^)	21 (20, 24)	25 (20, 28)	0.001
Peak airway pressure (cm H_2_O)	22 (19, 24)	19 (16, 21)	<0.001
Driving pressure (cm H_2_O)	21 (17, 23)	13 (10, 15)	<0.001
EtCO_2_ (mmHg)	46 (38, 56)	53 (44, 62)	0.003
PaO_2_ (mmHg)	350 ± 102	356 ± 118	0.773
PaCO_2_ (mmHg)	53 ± 10	63 ± 17	0.001
Hemoglobin (g/L)	106 (99, 115)	104 (99, 116)	0.752
PH values	7.24 ± 0.06	7.18 ± 0.09	<0.001
T6 Tidal volume (mL)	136 ± 50	84 ± 27	<0.001
Respiratory rate (min^−1^)	20 (18, 22)	23 (20, 25)	<0.001
Peak airway pressure (cm H_2_O)	20 (17, 21)	16 (15, 20)	<0.001
Driving pressure (cm H_2_O)	19 (16, 20)	10 (9, 13)	<0.001
EtCO_2_ (mmHg)	36 (32, 41)	44 (38, 52)	<0.001
PaO_2_ (mmHg)	388 (295, 427)	345 (298, 389)	0.104
PaCO_2_ (mmHg)	42 (39, 48)	50 (42, 56)	0.010
Hemoglobin (g/L)	106 ± 12	105 ± 11	0.467
PH values	7.29 ± 0.05	7.23 ± 0.03	<0.001

**Table 4:** Postoperative pulmonary complications.

Complications	Control Group n = 48 (%)	LPV Group n = 48 (%)
Pneumothorax	6 (12.5%)	0
Respiratory tract infections	10 (20.8%)	7 (14.6%)
Atelectasis	0	0
Postoperative hypoxemia	0	0
Total complications	17(33.3%)	7(14.6%)

## Data Availability

The datasets used and/or analyzed during the current study are available from the corresponding author on reasonable request.
